# Examining the relation of personality factors to substance use disorder by explanatory item response modeling of DSM-5 symptoms

**DOI:** 10.1371/journal.pone.0217630

**Published:** 2019-06-13

**Authors:** Fu Chen, Hongmei Yang, Okan Bulut, Ying Cui, Tao Xin

**Affiliations:** 1 Faculty of Psychology, Beijing Normal University, Beijing, China; 2 Department of Educational Psychology, University of Alberta, Edmonton, AB, Canada; 3 Collaborative Innovation Center of Assessment toward Basic Education Quality, Beijing Normal University, Beijing, China; University of New South Wales, AUSTRALIA

## Abstract

This paper explores how personality factors affect substance use disorders (SUDs) using explanatory item response modeling (EIRM). A total of 606 Chinese illicit drug users participated in our study. After removing the cases with missing values on the covariate measures, a final sample of 573 participants was used for data analysis. The *Diagnostic and Statistical Manual of Mental Disorders* (DSM-5) was used to measure the illicit drug users’ SUD level. Four personality factors–anxiety sensitivity, impulsivity, sensation seeking and hopelessness–along with gender and alcohol use were included in EIRM as person covariates. The results indicated that gender, alcohol use, and their interaction significantly predicted the SUD level. The only personality factor that strongly predicted the SUD level was sensation seeking. In addition, the interaction between gender and hopelessness was also found to be a significant predictor of the SUD level, indicating that the negative effect of hopelessness on SUD is stronger for women than for men. The findings suggest that sensation seeking plays an important role in influencing SUDs, and thus, it should be considered when designing intervention or screening procedures for potential illicit drug users. In addition, several DSM-5 SUD symptoms were found to exhibit differential effects by gender, alcohol use, and personality factors. The possible explanations were discussed.

## Introduction

Substance abuse and dependence is an increasing worldwide public health issue [1]. Investigating the influential factors of substance use problems is of great importance as these factors are helpful for the diagnosis, treatment, and prevention of substance use disorders (SUDs). Currently, the SUD diagnosis in the US and elsewhere is mainly based on the fifth edition of *Diagnostic and Statistical Manual of Mental Disorders* (DSM-5) [2], which is a standardized assessment used for diagnosing and classifying mental disorders. To date, a large body of studies applied item response theory (IRT) models to analyze DSM-5 SUD criteria across different populations and contexts. Despite the previous supportive findings for the validity of DSM-5 SUD criteria, the IRT approach was limited to psychometric analyses that only evaluated the measurement properties of individual DSM-5 SUD criteria, followed by separate statistical analyses (e.g. ANOVA or regression) to examine the relationship of SUDs and other relevant measures such as drug users’ background and personality variables. In this study, we apply the explanatory IRT modeling framework to analyze the DSM-5 SUD criteria by incorporating the illicit drug users’ personality factors, gender, and alcohol use as predictors of the SUD level. The combination of measurement modeling and statistical analyses within the model is expected to provide more comprehensive and unbiased validity evidence for DSM-5 SUD criteria. In addition, the psychometric property of each DSM-5 SUD criterion can be further examined by differential item functioning (DIF) analysis using person covariates in the model.

### Personality factors and substance use

Personality factors are often considered as strong indicators of individual differences in susceptibility to substance reinforcement in the previous theoretical framework of substance use (e.g. [3, 4]). In earlier ages, a large body of studies were interested in the relationships between personality factors of the five-factor model (FFM; e.g. [5]) and substance use problems. The FFM personality factors include openness to experience, extraversion, neuroticism, agreeableness, and conscientiousness. Many of these studies have revealed that FFM personality factors were influential for alcohol use. For example, it was consistently found that high neuroticism, low agreeableness, and low conscientiousness were significantly associated with alcohol use problems (e.g. [6–9]). In a meta-analysis examining the relationships between FFM personality factors and alcohol use using the cross-sectional data from 20 studies [10], it was found that individuals with severe alcohol use problems often showed high neuroticism, low agreeableness and low conscientiousness, consistent with findings of other meta-analysis studies (e.g. [11, 12]). Regarding illicit drug use problems, most previous studies have identified the same influential FFM personality factors. For example, [13] found that women’s drug craving was negatively related to conscientiousness and agreeableness. [14] found that compared with non-clinical participants, opioid-dependent individuals showed higher neuroticism, lower extraversion and lower conscientiousness, but similar levels of openness to experience and agreeableness. Similarly, in [15], cocaine/heroin users were found to show very high neuroticism and very low conscientiousness, and marijuana users were found to show high openness to experience, and low agreeableness and conscientiousness. In summary, regardless of the drug types, high neuroticism and low conscientiousness were found to be the common personality traits shown by users with problematic substance use across different studies [11].

Despite these broader FFM personality factors being well-examined, researchers were also interested in which non-overlapping specific personality facets were related to increased substance use. In previous studies, it was believed that individuals with different personality traits might show differences in susceptibility to the reinforcement effects of using alcohol and drugs [16]. According to a strand of studies in this theme (e.g. [17, 18]), their theoretical reasoning and empirical evidence suggested that increased substance use can be attributable to two different reinforcement processes, the positive reinforcement of substance use for a hedonic purpose and the negative reinforcement of substance use for relieving negative affective status. As such, the effect of personality factors on substance use might be mediated by these two types of reinforcements.

For example, anxiety and depression proneness were identified as individuals’ personality factors influencing their susceptibility to the negative reinforcement effects of using alcohol and drugs as a way to cope with adversities. More specifically, individuals with high anxiety proneness were more likely to seek for alcohol or anxiolytic substances to reduce the tension or other negative affect (e.g. [19, 20]), but less likely to use stimulants such as marijuana (e.g. [21]). Consistent with anxiety proneness, depression proneness was also found to be associated with alcohol use. For example, in a meta-analysis of eight longitudinal studies [22], it was found that depression predicted alcohol consumption quantity and vice versa. Moreover, it was found that depression-sensitive individuals were more likely to use analgesic drugs like opiates [23]. This is because depressive individuals would feel less pain due to affective and social problems by the inhibitory effects of using analgesic drugs or alcohol [5].

Regarding the positive reinforcement of substance use, impulsivity and sensation seeking were found to be strongly associated with substance use. For example, it was found that impulsivity-related personality factors were associated with problematic alcohol or drug use in both adolescents [17, 24, 25] and adults [26]. A recent review study also suggested that generally impulsivity was related to alcohol use, but it pointed out that different dimensions of impulsivity showed different degrees of correlation with the quantity and frequency of alcohol use, drinking problems and alcohol dependence [27]. Therefore, different impulsivity traits would affect the increased alcohol use in different ways [28]. With respect of sensation seeking, individuals with high sensation seeking were found to show elevated nicotine, alcohol, and marijuana use [29] as well as their elevated motivations for substance use [18, 30]. However, it should be noted that although whether impulsivity and sensation seeking indicate a single personality factor is controversial [31], they were found to show distinct mechanisms of influencing substance use [32], and therefore individuals with elevated impulsivity and sensation seeking should receive tailored prevention and intervention programs.

In summary, anxiety proneness, depression proneness, sensation seeking, and impulsivity were found to be four major influential personality factors of substance use in previous studies. The Substance Use Risk Profile Scale (SURPS) [33] further clarified these personality factors as follows: anxiety sensitivity, hopelessness, impulsivity, and sensation seeking. The validity of SURPS has been well investigated in both adults and adolescents and in different cultural contexts (e.g. [34, 35]).

### Explanatory item response modeling

A lot of studies applied the IRT framework to DSM-5 SUD criteria by examining their severity, discrimination, and item information (e.g. [36–38]). Notably, IRT analysis has been predominant for detecting the measurement invariance of DSM-5 SUD symptoms at the item level (e.g. [36]), which is called differential item functioning (DIF) analysis.

A typical IRT analysis employs a particular item response model to examine the relationships between examinees’ responses to items and a latent construct. For unidimensional IRT models, a fundamental assumption is that a “causal” common latent variable (e.g. a disorder) influences the responses to items (e.g. criteria). As such, an individual’s responses can be used to reflect his or her position on the underlying disorder continuum. IRT models use different functional forms to link persons’ levels on disorder continuum to the probabilities of endorsing criteria. A commonly used IRT model for dichotomously scored criteria is the Rasch model [39], in which the probability of the endorsement of person *j* on symptom *i* can be expressed as follows:

$$P(Y_{ij}=1)=\frac{\exp\left(\theta_{j}-\beta_{j}\right)}{1+\exp\left(\theta_{j}-\beta_{j}\right)}.$$ (1)

In Eq 1, *θ*_*j*_ denotes the person *j*’s level on the disorder continuum and *β*_*i*_ is used to indicate the criterion severity, which is defined as the location on the disorder continuum where the probability of endorsing a criterion is 0.5 (i.e. 50%). Higher severity parameter values mean that higher disorder levels are required for the criteria endorsement. Using the Rasch model, each individual’s latent disorder level can be estimated. This is advantageous to using sum or mean score as the disorder severity measure because each criterion’s different contribution to the estimation of latent disorder level can be identified by IRT.

Despite the advantages of traditional IRT analysis, it was often used to reveal the psychometric properties or establish the measurement models of instruments. In most substantive areas, however, how to explain the individual differences on item responses or latent construct is of great interest to practitioners and researchers. The explanatory IRT models are established based on the generalized linear mixed model (GLMM), in which item predictors, person predictors, and their interactions can be included as covariates in the same model. Therefore, the explanatory IRT models can integrate the measurement analysis and the statistical analysis for an explanatory purpose within a single model [40].

Using GLMM, Rasch model can be reconstructed in the following way: *η*_*ij*_ = *θ*_*j*_+*β*_*i*_+*ε*_*ij*_, in which *β*_*i*_ indicates the opposite meaning of symptom severity (the higher the value is, the easier the symptom endorsement will be), *θ*_*j*_ has a normal distribution with a mean of 0 and a variance of 1, and *ε*_*ij*_ has a normal distribution with a mean of 0 and a variance of π^2^ / 3. The logit link is used to transfer the probability of endorsement into a continuous scale (*η*_*ij*_) where

$$\eta_{ij}=ln(\frac{P(Y_{ij}=1)}{1-P(Y_{ij}=1)}).$$ (2)

As such, item predictors can be incorporated into the model as follows:

$$\eta_{ij}=\theta_{j}+\sum_{k=0}^{K}\beta_{k}X_{ik},$$ (3)

where *X*_*ik*_ indicates the value of item *i* on item property *k* (*k* = 1, …, *K*). It should be noted that in the above expression, the individual item parameter *β*_*i*_ is replaced by the linear combination of item properties (e.g. item categories), so the influence of item properties on responses can be represented by their fixed effects estimated by the model. This model is also called “Linear Logistic Test Model”, which allows interaction between different properties of items as well. Similarly, the person properties can be incorporated into Rasch model as follows,

$$\eta_{ij}=\theta_{j}+\beta_{i}+\sum_{p=0}^{P}J_{p}Z_{pj},$$ (4)

where *Z*_*pj*_ indicates the value of person *j* on person covariate *p* (*p* = 1, …, *P*), and the influence of each person covariate on item responses can be represented by the fixed effect *J*_*p*_. It should be noted that *θ*_*j*_ in the above expression indicates the remaining person effect accounting for the effects of person covariates. This model is also called “Latent Regression Rasch Model”. Moreover, the interaction between item and person covariates can also be incorporated into the Rasch model as follows,

$$\eta_{ij}=\theta_{j}+\beta_{i}+\sum_{h=0}^{H}\delta_{h}W_{ijh},$$ (5)

where *W*_*ijh*_ indicates the value of person *j* on the interaction between item *i* and a person covariate *h*. The influence of the interaction can be represented by the fixed effect *δ*_*h*_. This model can be used to detect DIF if the fixed effect of interaction is significant, which means the item functioning differs between different subgroups (e.g., males and females) given a certain person covariate (e.g. gender). In addition, the interaction between an item covariate and a person covariate can also be incorporated into the same model, which can be used to detect the differential facet functioning.

Obviously, the explanatory item response modeling (EIRM) differs from the traditional item response modeling in that the item and person covariates used for explanatory purposes can be modelled together within the IRT framework. In a typical two-step approach, the measurement model defined by IRT analysis only plays a role in the first step and the outcome variable based on IRT analysis is used in following separate statistical analyses for investigating the predictive effects of other relevant variables. However, using EIRM with person covariates, the group differences can be directly examined within the underlying measurement model. In other words, the differences in the latent disorder level can be parameterized at the group level, which leads to unbiased measures of group differences, differing from the typical two-step approach. For example, using the two-step approach, the expected a priori (EAP) estimates of participants’ latent construct score are firstly obtained by Rasch modeling, which are then used for regression analyses in the following steps. However, the EAP estimates have been shrunk toward the overall population mean by Rasch modeling. Therefore, the regression coefficients by the following analyses cannot reflect the conditional means of the subgroup populations. Additionally, larger measurement error leads to a higher extent to which the EAP estimates shrink to the population mean. As such, the regression coefficients of the two-step approach are largely influenced by measurement and estimation error [41].

### Current study

Several weaknesses of previous studies on DSM-5 SUD criteria can be identified. First, the question of how to investigate the influence of personality factors on SUDs was often addressed by a sequence of separate measurement and statistical analyses (e.g. [34]). However, this approach may lead to attenuated effects of personality factors because of the measurement errors of IRT analysis. Second, previous studies investigating the personality factors on substance use often used community samples rather than clinical samples (e.g. [35]). Indeed, a lot of previous studies on this topic used community samples given that recruiting community samples seems to be a standard practice in clinical research [42]. It was often believed that participants from the community samples show less severe substance use problems. Most previous studies in this area focused on how the personality factors of the five-factor model were related to substance use, and they often collected data from community samples such as adolescents and college students. For example, in some of their studies, alcohol use problems were found to be associated with high neuroticism and low agreeableness and conscientiousness [43, 44]. In contrast to studies using community samples, studies examining the effects of personality factors of the five-factor model by clinical samples were much rarer, but they demonstrated consistent findings for some non-drug substances like alcohol [45]. However, as we mentioned earlier, the roles of substance-use-related personality factors (i.e. anxiety sensitivity, hopelessness, impulsivity and sensation seeking) in influencing substance use problems were under-investigated. Although some empirical studies have revealed the effects of the four personality factors by community samples (e.g. [33, 34]), very few generalized these findings to clinical samples. A recent study found that the above four personality dimensions were sensitive factors among patients with alcohol and drug use problems [46], but it used a small inpatient sample and only half of the participants used illicit drugs. Therefore, to examine the generalizability of these findings, a clinical sample with illicit drug use problems is still needed.

This study aims to investigate the relation of personality factors to SUDs using the EIRM approach. The main purposes of this study are two-fold: the validity of DSM-5 SUD symptoms can be further examined by a clinical sample of illicit drug users; how personality factors predict SUD can be identified by incorporating them into the measurement model. In addition, because gender is an influential demographic variable on substance use disorder [47], and DIF by gender was present for some DSM-5 SUD criteria, we include gender as a person covariate in this study. Moreover, due to the fact that alcohol abuse and dependence present much higher prevalence than drug abuse and dependence in the population, and this pattern is severer for men than women (see [48]), the alcohol use and its interaction with gender should also be considered as person covariates for clarifying the unique contribution of personality factors.

The three specific research questions addressed in the present study are as follows: (1) What are the psychometric properties of DSM-5 SUD criteria, and do they show DIF by each of the predictors? (2) Do illicit drug users have latent SUD levels differing between men and women, or drinkers and non-drinkers? (3) Do anxiety sensitivity, hopelessness, impulsivity, and sensation seeking predict illicit drug users’ latent SUD levels, and if they do, how they are related to gender and alcohol use?

## Methods

### Sample

A total of 606 illicit drug users diagnosed with SUDs participated in our study. They were all under treatment at two drug rehabilitation centers located in southwestern China. All participants responded to the paper-form questionnaire under the guidance of one trained administrator, and they were informed that they could quit at any time during the data collection process. As such, all data used in our study was collected by self-report. Written informed consent for participation was obtained from each participant. The research protocol was approved by the ethics review committee of Beijing Normal University.

For this study, 33 cases with missing data on the covariate measures were removed from the original dataset, and thus, a total of 573 cases were used for analysis. The ages of participants ranged from 16 to 62 (*M* = 28.94, *SD* = 11.08). Nearly 70% of the participants were male. 71% and 96% of the participants were drinking and smoking regularly before the treatment, respectively. Regarding their ethnic groups, 73% of the participants are Han Chinese and others are from other Chinese ethnic minorities.

It should be noted that participants in our study were dependent illicit drug users rather than recreational drug users. However, it was believed that the recreational use of substances like alcohol, cannabis and ecstasy might influence their dependence on other severe substances like heroin [49]. In our study, heroin and methamphetamine were two primary drugs used by participants with endorsement percentages of 65.79% and 61.08% respectively, and 35.78% of participants used at least two different illicit drugs (see Table 1).

**Table 1:** Distribution of drug categories.

Drugs	Percentage (%)	Number of drugs	Percentage (%)
Cocaine	2.27	1	64.22
Hallucinogens	5.76	2	20.94
Heroin	65.79	3	9.25
Marijuana	5.58	4	3.31
MAMA	2.44	5	1.05
Methamphetamine	61.08	6	0.70
Prescription medicine	11.52	7	0.52
Synthetic cathinones	0.52	8	—
Other drugs	4.19	9	—

### Measures

#### DSM-5 SUD criteria

A published Chinese-translated version of DSM-5 SUD criteria [50] were used to measure the SUD level. Participants were asked to respond to all 11 criteria with a dichotomously scored scale. They were required to evaluate their lifetime use of illicit drugs. The coefficient omega of the 11 criteria is 0.79, indicating good internal consistency.

#### Substance use risk profile scale

The personality factors relevant to substance use was measured by the 23-item Substance Use Risk Profile Scale (SURPS) [33], which is composed of four subscales: (1) Hopelessness, a depression-associated personality factor which negatively reinforces the drug or alcohol use (seven items, e.g. “I am content.”; (2) Anxiety Sensitivity, a personality factor motivating substance use by reducing the anxiety symptoms (five items, e.g. “It scares me when I'm unable to focus on a task.”); (3) Sensation Seeking, a personality factor associated with thrill-seeking and the desire for stimulating, mood-enhancing effects of substance use (six items, e.g. “I would like to skydive.”); and (4) Impulsivity, a personality factor related to heavier, unconstrained drug use due to a minimal tolerance for negative emotions and rapid response to rewards of substance use (five items, e.g. “Generally, I am an impulsive person.”). The SURPS items were rated on a four-point Likert scale, six of which were inversely scored. The background variables including age, sex, smoking and alcohol use, and ethnicity were also measured.

The SURPS scale used in our study was a Chinese-translated version of the original scale. To make the instrument conceptually equivalent and perform in the same way in the Chinese context, we adopted the approach of forward-translations and back-translations to minimize the measurement bias. Specifically, two translators who were clinical psychology professionals and were also familiar with the terminology of SUDs, were invited to conduct the translation. Thereafter, an expert panel of the original translators and another two clinical psychology professors reviewed the initial translations and address the inadequacy of expressions and any discrepancies in the forward translation, which resulted in a complete Chinese-translated version of SURPS. Finally, the instrument was translated back to English by two researchers living in the English-speaking countries and familiar with the English-language culture. They were asked to focus on the conceptual and cultural equivalence of the instrument. Any discrepancies in the back-translation were further addressed by the expert panel, which resulted in the final version of Chinese SURPS.

To evaluate the psychometric properties of SURPS, we conducted the unidimensionality examination for each personality factor of SURPS given that the four personality factors were analyzed separately in our study (see Table 2). The comparative fit index (CFI), the Tucker Lewis index (TLI), the root mean square error of approximation (RMSEA), and the standardized root mean square residual (SRMR) were used as the model-data fit indices. According to previous studies [51, 52], the following cut-off values for these indices were used to indicate an acceptable fit: above 0.90 for CFI and TLL, below 0.08 for RMSEA and SRMR. We did not present the chi-square statistics because it was found to be too sensitive to large samples [53]. According to the model-data fit indices, the personality factor of hopelessness could obtain a good fit only if the the third and the seventh indicators were set to be correlated with each other. These two items both describe participants’ positive attitudes toward the future, which might be conceptually overlapping to some extent. The coefficient omega was used to indicate the internal consistency for each personality factor (see Table 2).

**Table 2:** Psychometric properties of personality factors.

Factor	*N*	CFI	TLI	RMSEA	SRMR	ω
Hopelessness	7	0.81	0.72	0.12	0.07	0.70
Hopelessness*	7	0.96	0.94	0.06	0.04	—
Anxiety Sensitivity	5	0.99	0.98	0.04	0.02	0.65
Sensation Seeking	6	0.93	0.89	0.07	0.05	0.65
Impulsivity	5	0.99	0.98	0.03	0.03	0.61

*N* refers to the number of items for personality factor. Hopelessness* indicates a modified model where the third and the seventh items are set to be correlated with each other.

### Data analysis strategy

First, the item parameter and participants’ latent scale scores of DSM-5 SUD symptoms were estimated by Rasch modelling without any person covariates. The exploratory factor analysis (EFA) was employed to check the unidimensionality of SUD criteria by the *psych* package in R [54]. Second, the person covariates predicting illicit drug users’ SUD level were investigated by the model comparison approach using EIRM. A null model without any person predictors would be firstly established, followed by increasingly complex models with more and more person predictors. The model-data fit of the new model was compared to the previous one. Because the previous models are nested within the new models, a likelihood ratio (LR) test can be used for model comparison. A significant LR test statistic indicates the improvement in goodness of model-data fit. At last, to detect the DIF symptoms, the interactions between item and each person predictor were included in EIRM. The EIRM analyses were conducted using the *lme4* package in R [55].

## Results

### Rasch model

**Fig 1:**
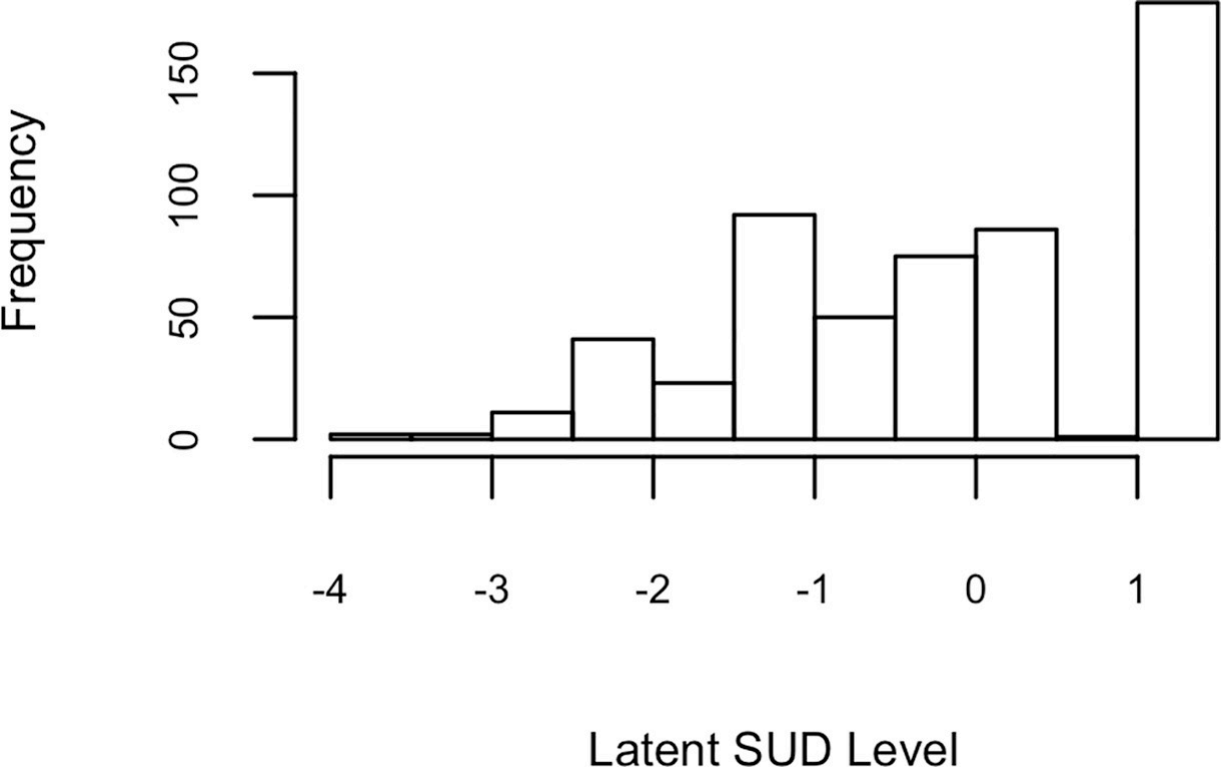
Histogram of participants’ latent scale score of SUD by the Rasch model.

Regarding the unidimensionality assumption, the EFA results show that the first five largest eigenvalues of factors are 4.88, 1.01, 0.24, 0.12, and 0.01, and one-factor solution accounts for 44% of the data variance. In addition, according to the scree plot, the eigenvalues drop largely from the first factor to the second factor. Moreover, the factor loadings of SUD criteria range from 0.44 to 0.82. Accordingly, it can be concluded that the illicit drug users’ responses to SUD criteria are mainly influenced by a single factor. According to the results of Rasch modeling, all symptoms present negative severity parameter values (see Table 3) and the histogram shows that the distribution of latent scale scores of SUD is negatively skewed (Fig 1), indicating that most participants presented very high latent SUD level.

**Table 3:** Descriptive summary of the DSM-5 SUD criteria.

#	Short-code	Mean	*SD*	Prevalence (%)	Severity
1	Larger	0.69	0.46	68.94	−1.18
2	Cut down	0.81	0.40	80.63	−2.05
3	Time spent	0.77	0.42	76.61	−1.72
4	Craving	0.66	0.47	65.97	−0.99
5	Major role	0.81	0.39	81.33	−2.11
6	Social	0.85	0.36	84.64	−2.42
7	Give up	0.86	0.35	86.21	−2.58
8	Hazard	0.73	0.44	72.95	−1.46
9	Consistent use	0.85	0.35	85.34	−2.49
10	Tolerance	0.76	0.43	75.92	−1.67
11	Withdrawal	0.76	0.43	76.09	−1.69

“Severity” refers to the item difficulty parameter by Rasch model without any person covariates.

### EIRM

#### Null model

The persons were considered as random effects in the null model, which is the same as the Rasch model without covariates used in previous analysis.

#### Modelling main effects of gender and alcohol use

Model 1 was established by adding gender and alcohol use (see Table 4). The difference in deviance values between the null model and model 1 is significant (*p* < .01), therefore we could statistically infer that model 1 fit data better compared to the null model. Moreover, the fixed effects represented by latent regression coefficients show that both gender and alcohol use significantly predicted the latent SUD level. Specifically, compared to men, women have a lower likelihood of symptom endorsement and thus have lower SUD level (*p* < .05), and compared to drinkers, non-drinkers have lower latent SUD level (*p* < .01).

**Table 4:** Fixed effects and model comparison results from the explanatory IRT models.

		Null Model	Model 1	Model 2	Model 3	Model 4	Model 5
Intercept (variance)		2.56 (1.60)	2.47 (1.57)	2.38 (1.54)	2.34 (1.50)	2.19 (1.48)	2.21 (1.49)
Gender (Female)			−0.35* (0.17)	−0.53** (0.20)	−0.42** (0.20)	−2.92** (1.10)	−0.37(0.07) (0.21)
Alcohol use (No)			−0.50** (0.17)	−0.73*** (0.21)	−0.76*** (0.20)	−0.75*** (0.20)	0.22 (1.16)
Gender (Female)*Alcohol use (No)				0.88* (0.36)	0.92** (0.35)	0.89* (0.35)	0.87* (0.37)
Hopelessness					0.17 (0.13)	−0.06 (0.16)	0.15 (0.16)
Impulsivity					0.23 (0.14)	0.17 (0.16)	0.15 (0.17)
Sensation Seeking					0.55*** (0.14)	0.36*** (0.17)	0.70*** (0.17)
Anxiety Sensitivity					0.06 (0.15)	0.15 (0.18)	0.12 (0.17)
Gender (Female)/Alcohol use (No)*Hopelessness						0.66* (0.27)	−0.06 (0.27)
Gender (Female)/Alcohol use (No)*Impulsivity						0.24 (0.30)	0.32 (0.30)
Gender (Female)/Alcohol use (No)*Sensation Seeking						0.52 (0.29)	−0.49 (0.30)
Gender (Female)/Alcohol use (No)*Anxiety Sensitivity						−0.31 (0.31)	−0.20 (0.34)
AIC		5724.2	5715.3	5709.8	5686.6	5684.8	5691.0
BIC		5805.1	5809.8	5811.0	5814.8	5840.1	5846.2
−LL		5700.2	5687.3	5679.8	5648.6	5638.8	5645.0
*df*		12	14	15	19	23	23
LR test	Δ*df*		2	1	4	4	4
	Δ−LL		12.88**	7.48**	31.22***	9.74*	3.61

*** = *p* < .001

** = *p* < .01

* = *p* < .05. AIC = Akaike Information Criterion, BIC = Bayesian Information Criterion, LL = Log Likelihood. Standard errors are presented in parentheses below fixed effects.

#### Modelling interaction between gender and alcohol use

Next, it would be interesting to understand how the effect of alcohol use on the SUD level differs between men and women. This can be investigated by including the interaction between gender and alcohol use as a new predictor (model 2). The LR test between model 1 and model 2 shows that the difference in model deviance is significant (*p* < .01), indicating that model 2 fit the data better. The main effects of gender and alcohol use are still negatively significant (*p* < .01 and *p* < .001 respectively). The interaction effect is positively significant (*p* < .05), indicating that compared to men, women have higher SUD level for no alcohol use than alcohol use. In other words, alcohol use reinforced men’s SUD level, whereas alcohol use improved women’s SUD level.

#### Modelling personality factors

The four personality factors were added into model 3 as new person predictors. The LR test shows that the difference in model deviance is significant (*p* < .001), indicating that model 3 largely improves the goodness of model-data fit. In model 3, the main effects of gender and alcohol use and their interaction stay significant in the same direction as those in model 2 (*p* < .01, *p* < .001 and *p* < .01 respectively). Only the personality factor of sensation seeking significantly positively predicts the SUD level with the largest fixed effect. It should also be noted that impulsivity, although not statistically significant, has the second largest fixed effect with *p* value of 0.088, which may indicate that impulsivity has a non-negligible reinforcing effect on SUDs. For hopelessness and anxiety sensitivity, their fixed effects are both positive but not significant.

#### Modelling interaction between gender and personality factors

Compared to model 3, model 4 included four additional interaction terms between gender and each personality factor. The model comparison test shows that the improvement in goodness of model-data fit is significant (*p* < .05). In model 4, the effects of gender and alcohol use and their interaction again stay significant (*p* < .01, *p* < .001 and *p* < .05 respectively). Similarly, only the main effect of sensation seeking is found to be significant (*p* < .001). Regarding the interaction between gender and each personality factor, only the effect of gender by hopelessness is significant (*p* < .05), which indicates that the negative effect of hopelessness on SUDs is stronger for women than men.

#### Modelling interaction between alcohol use and personality factors

Model 5 is used to examine the interaction effects between alcohol use and each personality factor. It should be noted that model 5 would be compared with model 3 rather than model 4 as model 3 is nested within both model 4 and model 5. The model comparison test shows that the difference in model deviance is not significant (*p* = 0.46), which indicates that the inclusion of alcohol use by personality factors has no contribution to the improvement in goodness of model-data fit. In model 5, only the interaction between gender and alcohol use and the main effect of sensation seeking are found to be significant (*p* < .05 and *p* < .001).

### DIF analyses

The group comparison by person properties assumes that the DSM-5 SUD measure is not biased, and each symptom functions in the same way across different subgroups. The DIF analysis, which investigates whether an item demonstrates differential psychometric properties for different sub groups, is conducted to examine this assumption. In EIRM, it can be addressed by including the person by item interaction. For instance, a significant effect of gender by item means how the item contributes to the estimation of latent construct level differs between men and women, which is not expected due to its confounding influence on group comparison.

**Fig 2:**
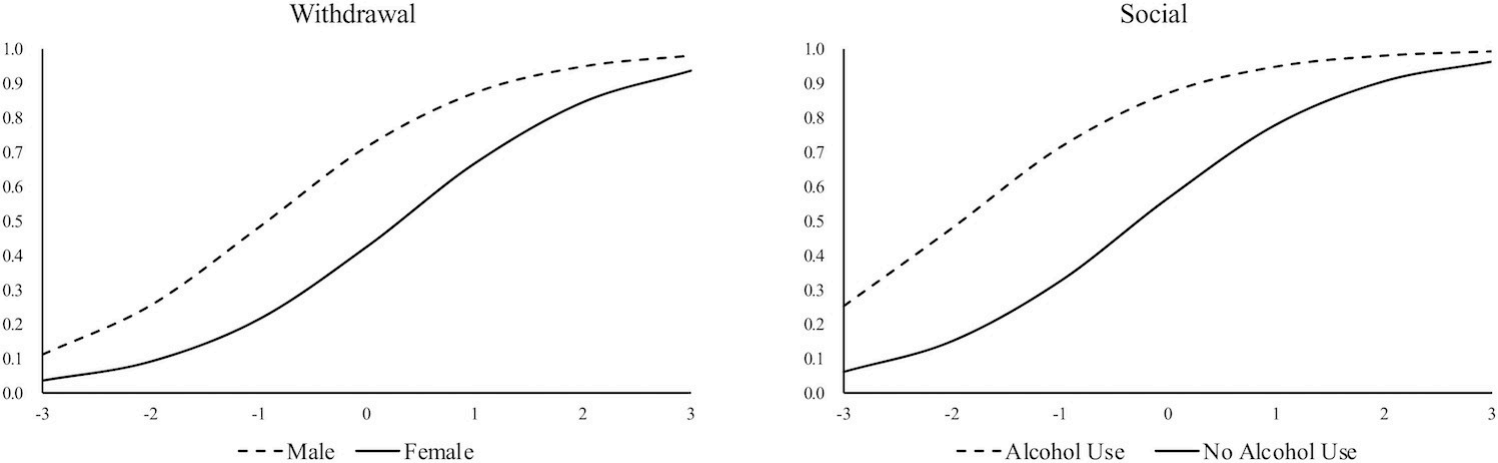
ICC plots of two example DIF symptoms. The left panel presents the ICC of “withdrawal”, and the right panel presents the ICC of “social”.

Table 5 shows the identified DIF items for each person predictor. Most DSM-5 SUD symptoms show DIF for gender. Specifically, compared to men, the symptoms “craving”, “hazard”, “tolerance” and “withdraw” are severer and thus harder to endorse for women, and the symptoms “give up” and “consistent use” are less severe and thus easier to endorse for women. The item characteristic curve (ICC) of symptom “withdrawal” is shown in Fig 2 as an example of visual presentation for DIF. According to Fig 2, given the same latent SUD level, men consistently have higher endorsement probabilities of symptom “withdrawal”, which indicates that this symptom functions differently between men and women. For the alcohol use, given the same SUD level, women have a lower likelihood of endorsing the symptoms of “social” and “give up” than men do. Another example ICC plot of symptom “social” is presented in Fig 2. Regarding the personality factors, generally, most DSM-5 SUD symptoms show no DIF. However, the symptom “social” shows DIF for impulsivity, which indicates that the symptom “social” is harder to endorse for illicit drug users with higher impulsivity levels. In other words, the “social” symptom is less common for highly impulsive illicit drug users. In addition, the symptom “give up” is harder to endorse and the symptom “tolerance” is easier to endorse for the illicit drug users with higher sensation seeking levels.

**Table 5:** Identified DIF items by each person predictor.

Symptoms	Gender (Female)	Alcohol use (No)	Hopelessness	Impulsivity	Sensation Seeking
Craving	−0.73* (0.32)				
Major role					
Social		−1.65*** (0.36)		−0.89** (0.28)	
Give up	1.00* (0.41)	−0.73* (0.36)			−0.62* (0.27)
Hazard	−0.74* (0.33)				
Consistent use	1.01* (0.41)				
Tolerance	−1.09*(0.33)				0.52* (0.25)
Withdrawal	−1.23*(0.33)				

*** = *p* < .001

** = *p* < .01

* = *p* < .05. Standard errors are presented in parentheses below fixed effects.

## Discussion

To our knowledge, this is the first study applying EIRM in clinical studies specific to SUDs. Our study contributes to the literature in several important ways. First, to investigate the effects of personality factors on SUDs, we used EIRM rather than the traditional two-step approach because of its advantages discussed previously in this paper. Second, the clinical sample used in this study is of great value for understanding the relation of personality factors to SUDs among illicit drug users. Third, the validity of DSM-5 SUD criteria has been further justified by the EIRM approach including personality factors in the measurement model. Fourth, at the individual symptom level, the DIF symptoms were identified by EIRM approach. Given the relation of personality factors to SUDs, the findings of this study should be beneficial for practitioners who make diagnoses and design interventions for illicit drug users.

Comparing the severity parameters of each individual symptom, we found that the newly added symptom “craving” was severest. This is in accordance with the finding that “craving” is one of the three severest symptoms [36]. This finding indicates that the “craving” symptom is relatively harder to be present in illicit drug users. In other words, the “craving” symptom may be problematic for other common substances due to its low prevalence. Some other studies also demonstrated that adding this symptom might be problematic (e.g. [38]) and did not contribute to more information in the measure [56]. The “social” symptom was found to be not as severe as other symptoms in this study, which is contrary to the previous finding [36]. This may be due to that the users’ social behavior was largely inhibited by the illicit drug use, but it would not be a concern for tobacco or alcohol use. That is to say, the same symptom may function differentially for different substances (e.g. [57]).

The EIRM analyses found that both gender and alcohol use play a significant role in explaining the individual differences in SUD levels. Specifically, compared to men, women presented lower latent SUD level. This finding is consistent with most previous findings that men suffer higher prevalence rates of SUDs (see [47]). A possible explanation of the gender difference is that women suffer more stigmatization than men for substance use [58]. Additionally, drinkers showed higher SUD level than non-drinkers did, which suggests that alcohol use may be a facilitator for illicit drug use, or alcohol users and illicit drug users share similar personality factors motivating the elevated SUDs. Moreover, it is interesting to note that how alcohol use predicts SUDs differs between men and women. That is, women with alcohol use showed lower SUD levels whereas the opposite for men. This can be explained by the fact that men and women have different negative consequences due to substance use. Previous studies revealed that compared to men, women suffer severer medical, physiological, and psychological impairment earlier in long-term alcohol use [47], which therefore inhibits their use of other substances.

Regarding the personality factors, stronger sensation seeking leads to higher SUD levels, which is consistent with previous findings that sensation seeking is a strong and independent predictor of substance use [59, 60] with the strongest prediction power among different personality factors [61]. However, the other personality factors were not associated with illicit drug use in this study. In previous studies, neuroticism-related personality factors were found to be strongly associated with SUDs (e.g. [11, 34, 59]). In our study, hopelessness and anxiety sensitivity, which could be considered as neuroticism personality facets, did not significantly predict illicit drug use. As we mentioned previously, anxiety sensitivity was found to be mostly related to alcohol or anxiolytic substance use, but not stimulant substance use (e.g. 19–21), and hopelessness was found to mostly related to analgesic drugs [23]. This is because these types of substances are often desired by users who wish to reduce their anxiety or affective pain levels. However, it should be noted that participants in our study mostly used heroin or methamphetamine, which were significantly different from alcohol or analgesic drugs in terms of their addictive levels and harms. It is therefore possible that neuroticism-related personality traits have little impact on severe illicit drug users. Regarding the finding that sensation seeking was the only predictive factor of illicit drug use, we provide the following possible explanations. First, compared with other personality factors, sensation seeking might be a more unique and dominant factor influencing substance use problems. In a study evaluating the psychometric quality of SURPS with a clinical sample of substance users [46], it was found that among the four SURPS factors, sensation seeking was the only one which was not significantly associated with any other personality measures, implying that sensation seeking shared no overlapping components with the other personality traits. Given that all personality factors were modelled simultaneously in our analysis, the identified prediction effects represent the unique contribution of each personality factor to illicit drug use. Therefore, after controlling for the effects of the other personality factors, some personality factors showed no significant regression coefficients. Second, sensation seeking might be more prominent for severe substance use problems. For example, it was found that sensation seeking did not predict alcohol use but strongly predicted drunk, marijuana or hallucinogens use [34], and individuals with strong sensation seeking were more likely to have heavy drinking and alcohol use problems [59]. In our study, unlike alcohol and cigarettes, heroin and methamphetamine are highly intense and addictive drugs, which cause much heavier symptoms than typical substances. Our finding suggested that sensation seeking might be the most important personality trait which differentiates illicit drug users from individuals with other substance use problems. Third, it is also possible that the cultural context also plays a role in the relations of personality factors to SUDs, since relevant research findings were not consistent in previous studies. For example, it was found that sensation seeking and impulsivity significantly predicted substance use problems in English, Irish, French, and German adolescents [62], but none of the four personality factors was found to be associated with substance use behaviors of adolescents in Hong Kong [35]. Despite this discrepancy being identified by adolescent samples, it is still possible that the relationship between sensation seeking and illicit drug use becomes stronger under the Chinese cultural context. However, more empirical comparative studies are needed to reveal how cultural differences influence the relation of personality factors to SUDs. At last, it is possible that personality factors excluding sensation seeking function as motivators for the illicit drug use at the initial stage and sensation seeking functions as a strong predictor of their elevated SUD levels at the later stages. In our study, the participants were all diagnosed with severe SUDs and most of them had long illicit drug use history. Therefore, it is possible that only sensation seeking was capable of predicting their drug use behaviors and symptoms. However, the other personality factors are still valuable for explaining why they used illicit drugs at the earlier life stages. This finding is of great practical implication in that interventions on sensation seeking may be more helpful for alleviating severe illicit drug users’ symptoms.

Furthermore, our study identified several DSM-5 SUD symptoms with DIF. Most of them were found to show DIF by gender. This may be attributable to gender differences in social pressure, biological response, and medical consequences between men and women [47]. For example, because women are more likely to be stigmatized for substance use, they are thus more likely to give up social, occupational, or recreational activities, which may lead to DIF associated with the symptom of “give up”. In addition, the same biological response is induced by smaller quantities of substances for women than for men, which may introduce DIF for the symptoms of “tolerance” and “withdrawal”. The “social” symptom showed DIF by alcohol use. This may be due to that drinking behavior is strongly motivated by social demands under Chinese culture. For non-drinkers, they may have fewer social activities and therefore have fewer social or interpersonal problems. As such, the “social” symptom may show higher severity for non-drinkers. A similar explanation also applies to illicit drug users with high impulsivity. For illicit drug users with a strong sensation seeking, it is possible that stronger sensation seeking leads to a stronger desire for illicit drugs, so they are more likely to present “tolerance” symptom. Despite these possible explanations, the causes for DIF should be further scrutinized by clinical psychologists with content expertise and clinical experiences.

Some limitations exist in this study. First, the sample is imbalanced in terms of gender and alcohol use in this study. However, we suspect that the gender and alcohol use composition of our sample may well reflect that of the illicit drug user population in China. Our results therefore should be trustworthy. Second, it would be interesting to compare how the psychometric properties of DSM-5 SUD criteria, the influence of gender, alcohol use, and personality factors, and the presence of DIF differ when analyzing data from other substance users. Future studies are needed to cross validate our results with other samples. Third, although EIRM can be used to detect DIF symptoms, it cannot be used for the analysis of nonuniform DIF because EIRM is based on Rasch modeling in which the discrimination parameters of symptoms are fixed to be the same. Other DIF procedures like logistic regression are recommended to explore the presence of nonuniform DIF. At last, given that many participants presented multiple drug use (e.g., using heroin and methamphetamine simultaneously, see Table 1), we did not examine the specific relations of different personality factors to different types of illicit drugs. However, it would be interesting to examine the relationships between personality factors and drug types in future studies using homogeneous samples.
